# Crustal processes sustain Arctic abiotic gas hydrate and fluid flow systems

**DOI:** 10.1038/s41598-020-67426-3

**Published:** 2020-06-30

**Authors:** K. A. Waghorn, S. Vadakkepuliyambatta, A. Plaza-Faverola, J. E. Johnson, S. Bünz, M. Waage

**Affiliations:** 1 CAGE – Centre for Arctic Gas Hydrate, Environment and Climate, Department of Geosciences, UiT – The Arctic University of Norway, Dramsveien 201, 9037 Tromsø, Norway; 2 Department of Earth Sciences, University of New Hampshire, 56 College Road, Durham, NH 03824 USA

**Keywords:** Structural geology, Geophysics, Tectonics

## Abstract

The Svyatogor Ridge and surroundings, located on the sediment-covered western flank of the Northern Knipovich Ridge, host extensive gas hydrate and related fluid flow systems. The fluid flow system here manifests in the upper sedimentary sequence as gas hydrates and free gas, indicated by bottom simulating reflections (BSRs) and amplitude anomalies. Using 2D seismic lines and bathymetric data, we map tectonic features such as faults, crustal highs, and indicators of fluid flow processes. Results indicate a strong correlation between crustal faults, crustal highs and fluid accumulations in the overlying sediments, as well as an increase in geothermal gradient over crustal faults. We conclude here that gas generated during the serpentinization of exhumed mantle rocks drive the extensive occurrence of gas hydrate and fluid flow systems in the region and transform faults act as an additional major pathway for fluid circulation.

## Introduction

Intersections between Mid-Ocean Ridges (MOR) and Oceanic Transform Faults (OTF) are complex tectonic settings, where extensional and strike-slip tectonics meet. Inside corner highs form either at the Ridge-Transform Intersection (RTI) or at non-transform offsets along spreading ridges and are commonly topographically higher compared to the surrounding seafloor^1^. Generally, these features are coincident with crustal scale detachment faults, which can exhume ultra-mafic mantle rocks and accommodate spreading at slow and ultraslow, melt-poor spreading centers^2,3^. Proximity of an inside corner high to the spreading ridge, as well as comparative steepness^4^ can indicate whether the underlying faults are actively exhuming mantle material; areas of actively exhuming detachment faults are generally warmer, more buoyant and steeper than inactive or paleo-inside corner highs^1^. Fault splays and damage tips are also common at RTI’s, as RTI’s represent the juncture between two different faulting regimes^5^. RTI’s generally exhibit many different types of splay faulting, where the specific mode may vary depending on the kinematics of the moving blocks and relative angles between the main faults^5^.

MOR’s and OTF’s are often associated with increased fluid flow, heat flow and increased tectonic activity compared to the surrounding older, colder basement or continental margins. Deep fluid flow and seawater cycling are often higher in spreading ridge settings, constituting biogeochemical exchange between oceans and the solid earth^6^. Hydrothermal vent systems are common on spreading ridges, either hosted on crustal scale detachment faults (peridotite hosted) or associated with magmatic centers. Hydrothermal systems in peridotite hosted environments commonly vent abiotic methane^7–12^, a form of methane produced inorganically either through gas–water–rock interactions or magmatic processes^11^. Gas hydrates–crystalline compounds formed under high pressure and low temperature^13^—are inferred from geophysical data in marine sediments on the inside corner of a ridge transform intersection in the Fram Strait^14,15^. Johnson et al.^14^ and Waghorn et al.^15^ observe a close relationship between crustal detachment faults and the overlying gas hydrate system and hypothesize that abiotic methane produced from serpentinization reactions within fault zones and/or in fluid inclusions^16^ in ultramafic mantle rocks may contribute methane to the overlying gas and gas hydrate system. In many fluid flow settings, including hydrothermal systems^16^, hydrocarbon provinces^17^, mud remobilization and sand injectite systems^18^ and cold seep systems^19^, faults are considered important conduits for fluid migration in the subsurface. In addition, studies show that OTF’s are also able to host increased fluid flow and OTF’s may meet the prerequisites for abiotic methane production via serpentinization, where the OTF acts as conduits for seawater circulating to peridotites at depth^6^. Thermogenic methane forms at a depth where organic matter/kerogen is appropriately heated (> 150 °C) and under high pressure (usually between 2 and 4 km depth) to become methane^20^, while microbial methane forms when microbes in the shallow subsurface generate methane through the anaerobic oxidation of organic matter^21^. Generally, at the depth of most slow to ultraslow spreading mid-ocean spreading ridges, the pressure regime is appropriate for gas hydrate formation, however, most active mid-ocean spreading ridges are sites of increased geothermal heat, which may inhibit hydrate formation. Additionally, in well-developed ocean basins the active plate boundary is often sediment starved, which also prohibits hydrate formation and accumulation. Abiotic methane production and consequent venting at peridotite-hosted hydrothermal systems has been well-established^7,11,12,22–24^, however, it is still unclear whether abiotic methane is a contributing source to gas hydrate accumulations in settings where all the prerequisites for gas hydrate formation exist. Rajan et al.^25^ hypothesize the presence of abiotic methane on the eastern Knipovich Ridge flank, while Johnson et al.^14^ and Waghorn et al.^15^ hypothesize abiotic methane may contribute to the gas hydrate system on the Svyatogor Ridge on the western Knipovich Ridge flank. OBS velocity models in the area indicate that partially serpentinized mantle rock is present underlying the study area^26^. Sedimentary faults on the Svyatogor Ridge have developed due to movement on previously sedimented detachment faults^15^. The ultra-slow spreading, melt-poor Knipovich Ridge has an azimuth of ~ 308° and a half spreading rate (eastwards) at ~ 6–8 mm/year^27^. The strike-slip Molloy Transform Fault (MTF) is slightly oblique to the Knipovich Ridge^28^. Sedimentation on the western flank of the Knipovich ridge is dominated by contourites associated with the modern West Spitsbergen Current and its equivalent predecessor^14^.

**Figure 1:**
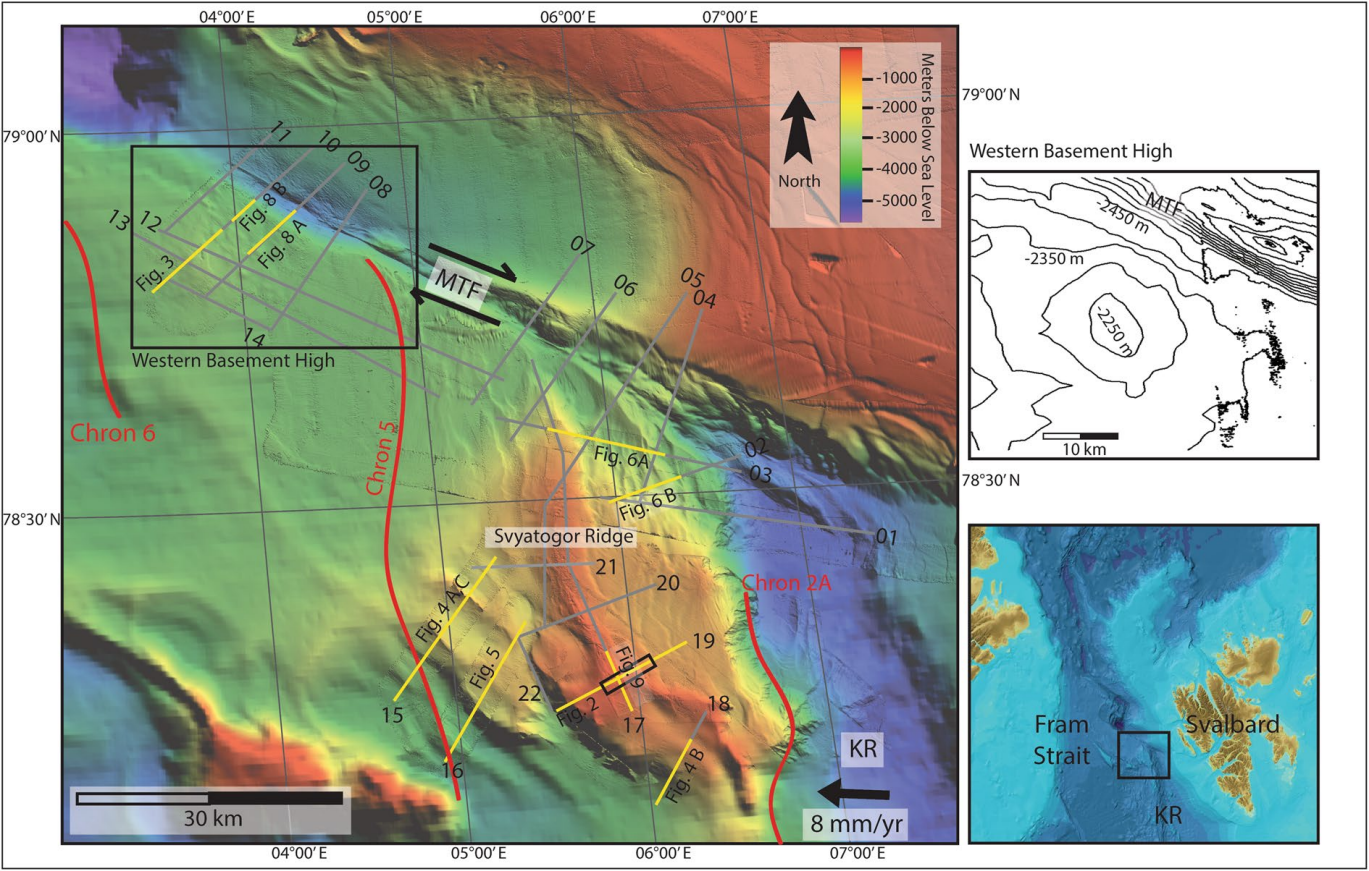
The study area and 2D seismic lines used in this work. Red lines denote magnetic anomaly chrons—Chron 6–19.6 Ma, Chron 5–9.8 Ma, Chron 2A—2 Ma^27^. The Knipovich Ridge (KR) separates the Eurasian plate from the north American plate, which is propagating westwards at about 8 mm/year^29^. The Molloy Transform Fault (MTF) denotes the northern extent of the RTI, and the seismic lines cover most of the inside corner high between the KR and MTF. Figures following in the text are denoted with yellow lines and the figure reference. The 3D seismic survey used in Waghorn et al.^15^ at the south of the Svyatogor Ridge is denoted with a black box, while line 19 is the seismic line also shown in Johnson et al.^14^.

This study presents evidence for extensive occurrence of gas hydrates and associated fluid flow systems in sediments on the inside corner of Knipovich Ridge MTF intersection and on the western flank of MTF (Fig. 1). We find that there is a strong correlation between fluid accumulations, crustal faulting and localized elevated geothermal gradients, indicating that active faulting on plate boundaries is responsible for gas hydrate and free gas accumulations here. Observations in this study area support the hypothesis^14^ that serpentinization reactions within the crust have produced abiotic methane, contributing to gas hydrate formation.

## Data and methods

We use 22 high-resolution 2D seismic lines and bathymetry data across the Molloy Transform Fault and the Knipovich Ridge collected 2014–2018 aboard RV Helmer Hanssen. The 2D streamer is 100 m long, consists 32 receivers and the seismic signal was produced using two mini GI airguns with volumes of 15/15 in^3^ and 30/30 in^3^. Processing of these seismic lines has been consistent across the surveys, using RadEXPro Software from DECO Geophysical, and consisted of; (1) Geometry assignment, (2) simple bandpass filter (20 Hz–30 Hz–300 Hz–350 Hz), (3) true amplitude correction, (4) despiking, (5) CDP binning at 3.125 m spacing, (6) Stacking and (7) Kirchhoff Migration using a manual velocity model based on CTD water velocity, and prior published velocity information in the area^26,30^. Interpretation used commercially available seismic interpretation software (Petrel).

The base of gas hydrate stability (BGHSZ) for each seismic profile was modelled using the theoretical phase boundary curve estimated using the CSMHYD program^13^. The program estimates multiphase equilibrium pressure values for a given temperature, with an error range of + /− 15%. The pressure estimates are converted to water depth assuming hydrostatic pressure. The three-phase temperature-depth curve is then compared with the subsurface thermal profile at each location on the seismic profile estimated using the measurements of bottom water temperature^31^ and geothermal gradient in the region^32,33^. Gas hydrates are considered stable at locations where the observed temperature is lower than the one predicted by the theoretical temperature-depth curve. Uncertainties in predicted BGHSZ depths for each seismic profile are estimated using uncertainties in observations of bottom water temperature, thermal gradient, and potential salinity variations for a MOR setting (Table 1). Salinity of pore water in the study area is unknown, with no measurements to provide insight. Salinity of deeply circulating fluids is highly variable, depending on factors such as temperature and pressure at depth of formation, rock properties and water–rock reactions^34^. To account for this, we established a range of 3–42 SU as extreme values of potential pore-water salinity. Although gas composition data does not exist for this site, we use two different gas compositions; (1) 100% methane and (2) 98% methane, 0.6% ethane and 0.4% propane (based on measurements at the Vestnesa Ridge^19,35^ to the north of the Svyatogor Ridge) to account for potential variations in the methane content. The base of gas hydrate stability in the seismic data is shown as a zone to highlight variations as a result of uncertainties in input parameters (Table 1) (Supplementary Figure 1).

**Table 1:** Parameters used for modelling the base of hydrate stability across the study area, and the error values where appropriate for developing a range of possible depths of the hydrate stability base.

Parameter	Range of value(s) used	Uncertainty
Bottom water temperature (°C)^31^	− 1	± 0.8
Geothermal gradient (°C/km)^32,33^	63–300	± 10
Porewater salinity (SU)	3–43	–
Gas composition (% methane)^14,35,36^	98–100	–

## Results

### Linking crustal scale tectonics with shallow sedimentary features

**Figure 2:**
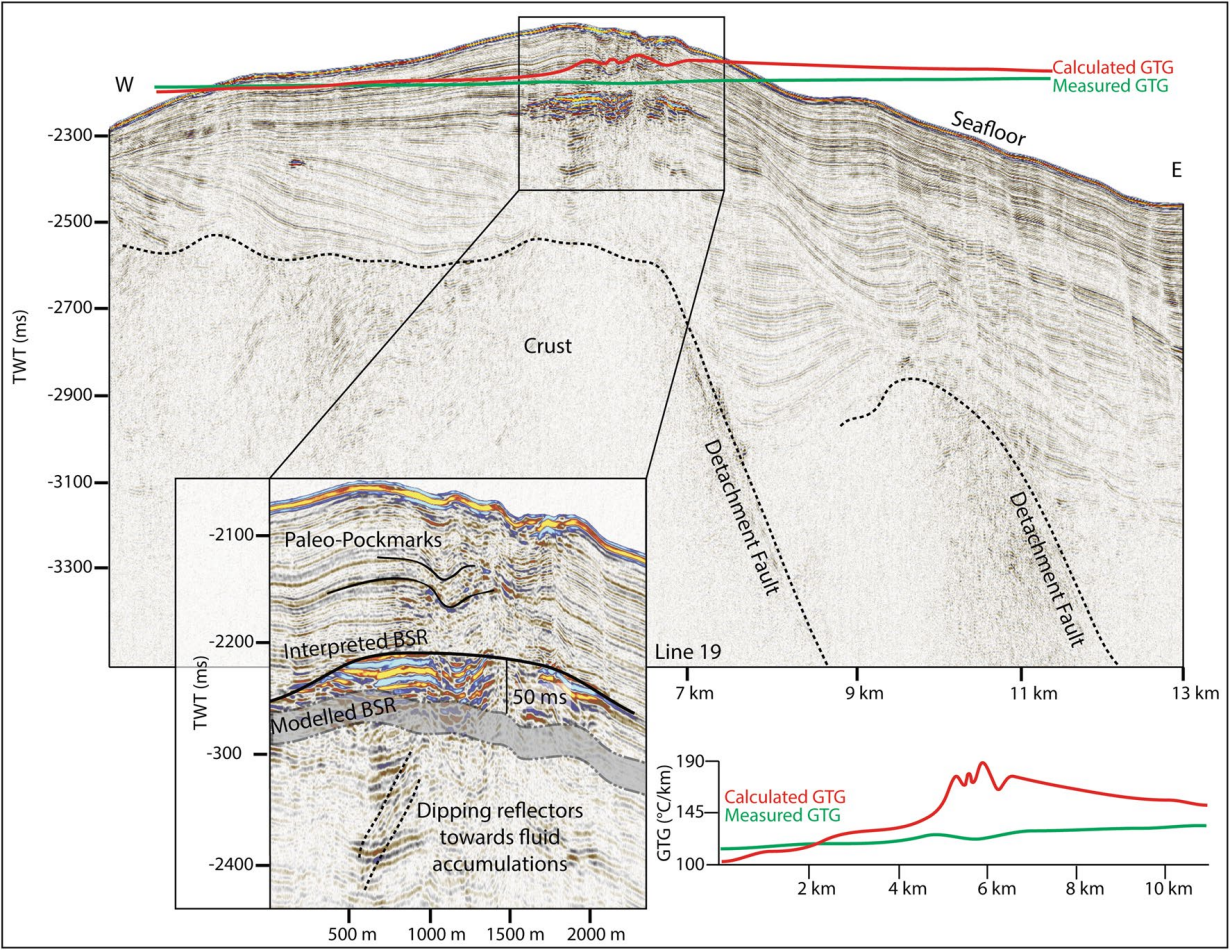
On line 19, basement structure and detachment faults are interpreted based on prior interpretations^14,15^. The upper reflection characterizing the basement is often undulating, while the internal reflections, where present, are chaotic. A reflection dipping approximately 30° pierces the basement structure in two places, interpreted as detachment faults. Above the western detachment fault is a BSR reflector approximately 60 ms above the modelled BSR. Above the BSR reflector are horizons of paleo-pockmarks interpreted by Waghorn et al.^11^, and seafloor pockmarks. We also identify reflections beneath the free gas zone dipping towards the fluid accumulation which crosscut the otherwise continuous strata. Geothermal gradients estimated from the depth of the observed BSR (red) are markedly higher than measured geothermal gradient^37^ values (green) particularly in the area with paleo-pockmarks.

**Figure 3:**
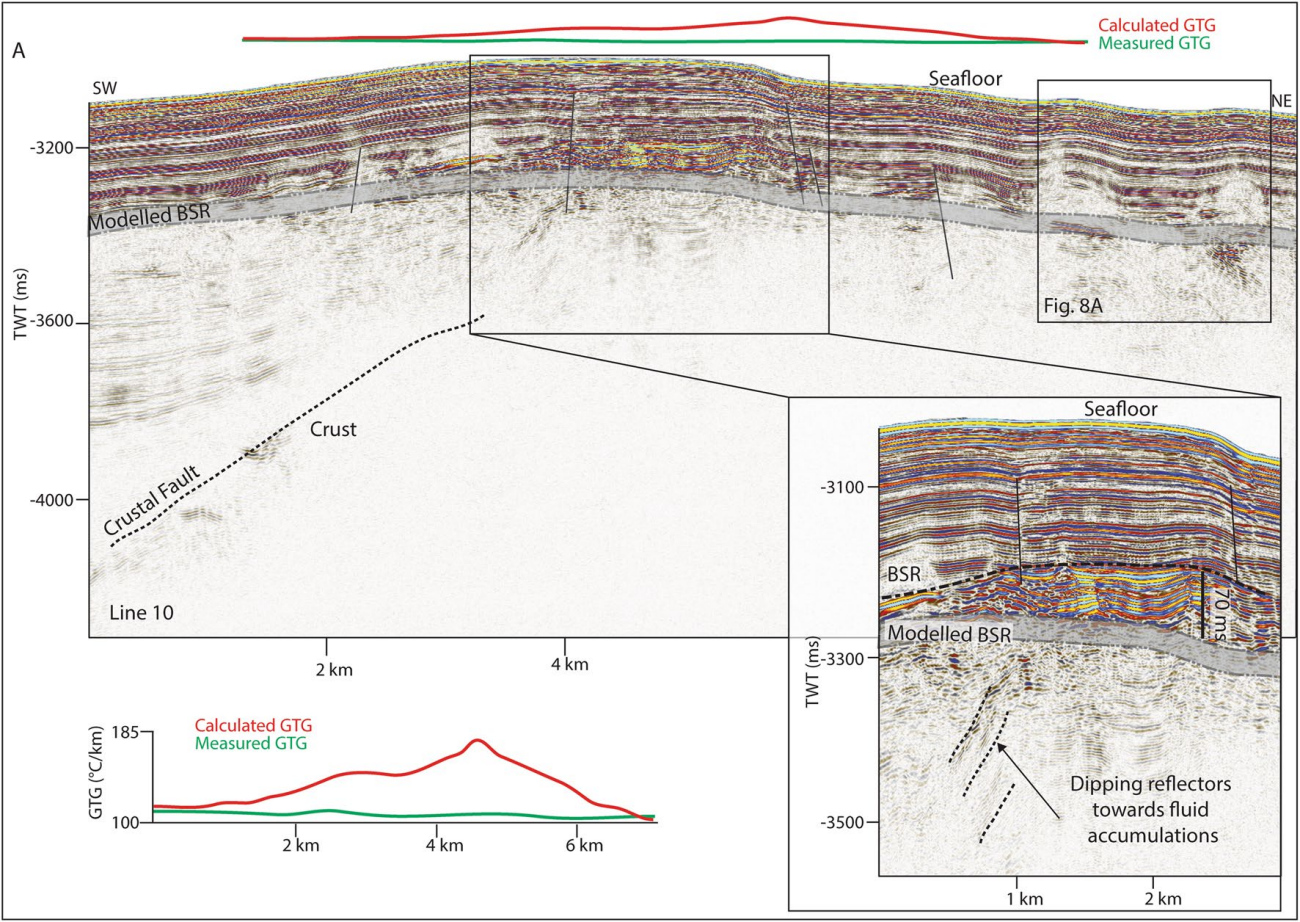
The northwestern extent of the study area has a small bathymetric high where we observe a BSR (70 ms above the modelled BSR) with reflections dipping upwards towards the free gas zone. Reverse polarity reflections interpreted as fluid accumulations (RPFA) are observed off the bathymetric high also with reflections dipping upwards towards them. These are also interpreted as free gas trapped beneath hydrate. Geothermal gradients estimated from the depth of the observed BSR (red) are markedly higher than measured geothermal gradient^37^ values (green) especially at the location of faults.

Here we use high-frequency seismic data and can identify chaotic reflections, with undulating or rough upper boundaries. These features are usually located at the limit of seismic energy, with no coherent reflections appearing beneath (Fig. 2) and are interpreted as the top of oceanic crust. Consistent with the interpretation by Johnson et al.^14^ and Waghorn et al.^15^ the oceanic crust in our study area is clearly recognized at a depth of ~ 500 ms below seafloor (bsf) on line 19 (Fig. 2). At the western end of the study area, bathymetric data shows a small ridge-like structure at the southern flank of the MTF (Fig. 1 inset). Seismic data there show chaotic, undulating reflections, and a quickly deteriorating seismic signal at ~ 200 ms below seafloor (Fig. 3). In correspondence to the eastern part of the study area, we interpret this also as an oceanic crustal high. One long semi-coherent reflection pierces the interpreted crust at an angle of approximately 30°, located directly beneath the high. This feature is tentatively interpreted as a crustal fault (Fig. 3) following Waghorn et al.^15^.

**Figure 4:**
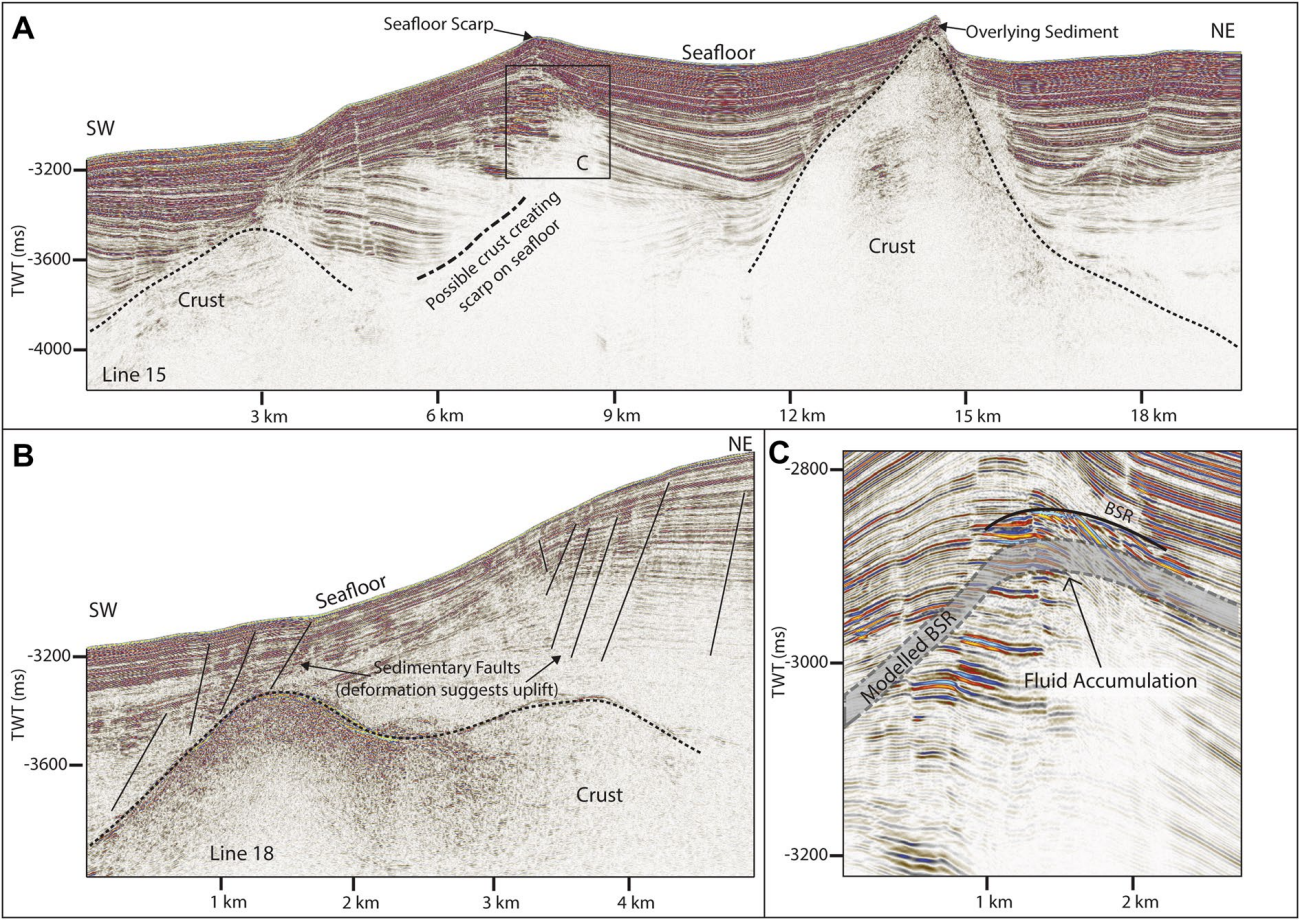
Lines 15 and 18. Here basement is again interpreted, consisting of large peaks on line 15 (**A**) and a steep flank with undulating top on line 18 (**B**). Reflector configuration and seafloor topography suggests that on line 15 (**A**) there is a third peak, however there is a fluid accumulation (**C**) which correlates to the location of the modelled BSR which is likely scattering seismic energy.

**Figure 5:**
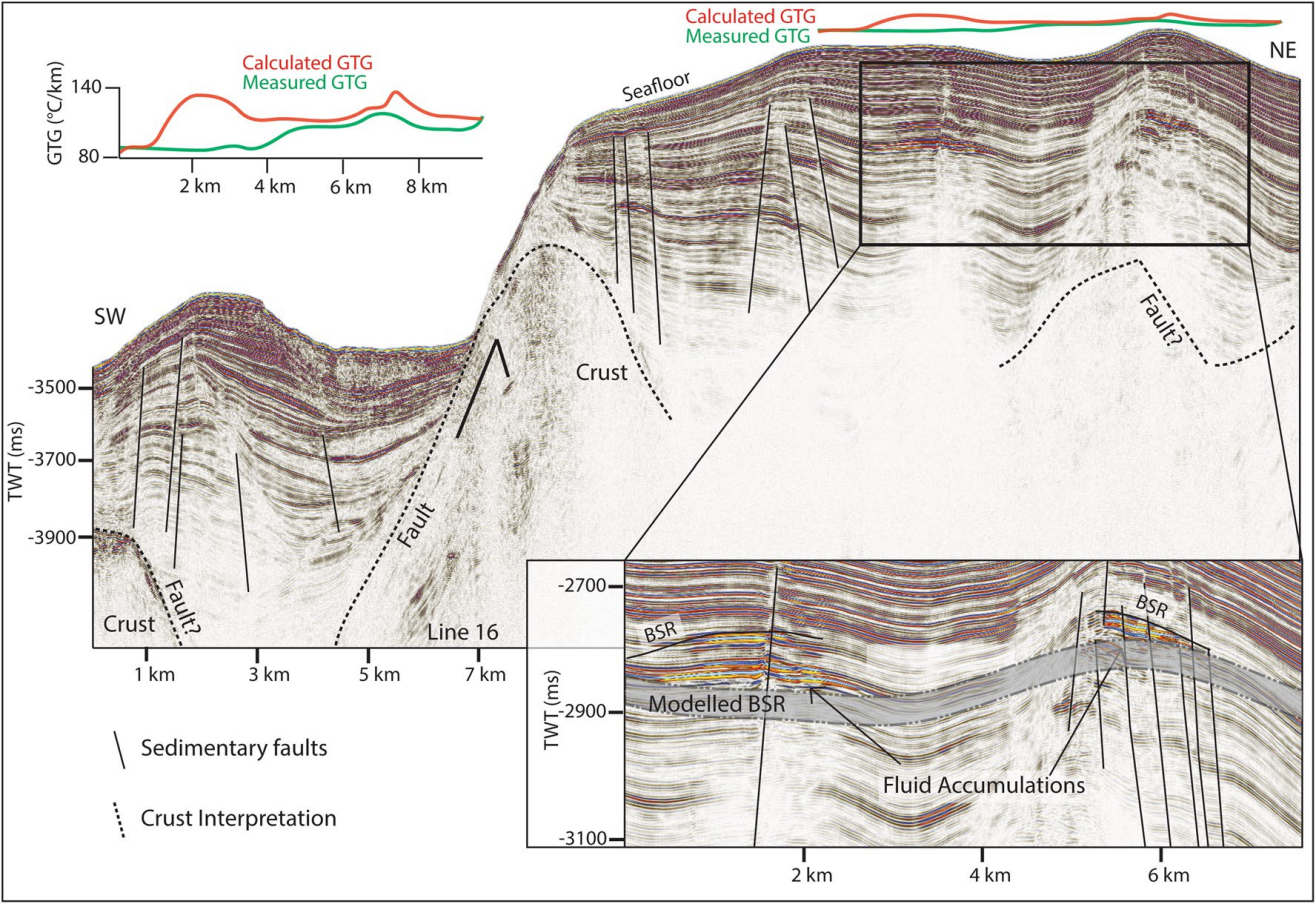
Line 16. Here we interpret three, potentially four, areas of crust with steep flanks creating a marked change in seafloor topography. In addition to the interpreted crust faults, the areas of crust outcrop correspond to areas of increased sedimentary faulting. The inset figure shows high amplitude anomalies cutting across sedimentary strata. These anomalies are interpreted as fluid accumulations beneath a BSR. However, the BSR occurs significantly above the predicted base of gas hydrate stability. Geothermal gradients estimated from the depth of the observed BSR (red) are markedly higher than measured geothermal gradient^37^ values (green) especially at areas with fluid accumulations and faults.

Southwest of the Svyatogor Ridge, correlating with a large scarp in bathymetry data we interpret a crustal high with steep, sediment covered flanks (Fig. 4A, B, 5). Due to the correlation of this scarp with other parallel scarps in bathymetric data (Fig. 1), we interpret these as additional basement crustal highs, likely faulted into their current position. The kinematics of these faults is unclear as the seismic lines are 2D; however, based on the extent of the scarps forming on the seafloor above, it appears they are normal faults dipping southwest at ~ 30° (Figs. 1, 5). In conjunction with this is a smaller scarp (Fig. 4A, C) which, although we do not image the crust beneath it, we interpret it to result from a crustal high and/or faulted crust. Based on the bathymetric map, these potential faults are not the same strike as the Knipovich spreading ridge fabric at Svyatogor Ridge, but instead more closely resemble the strike of the MTF (Fig. 1). In this region, Crane et al.^33^ interpret a paleo-transform fault with a similar strike.

**Figure 6:**
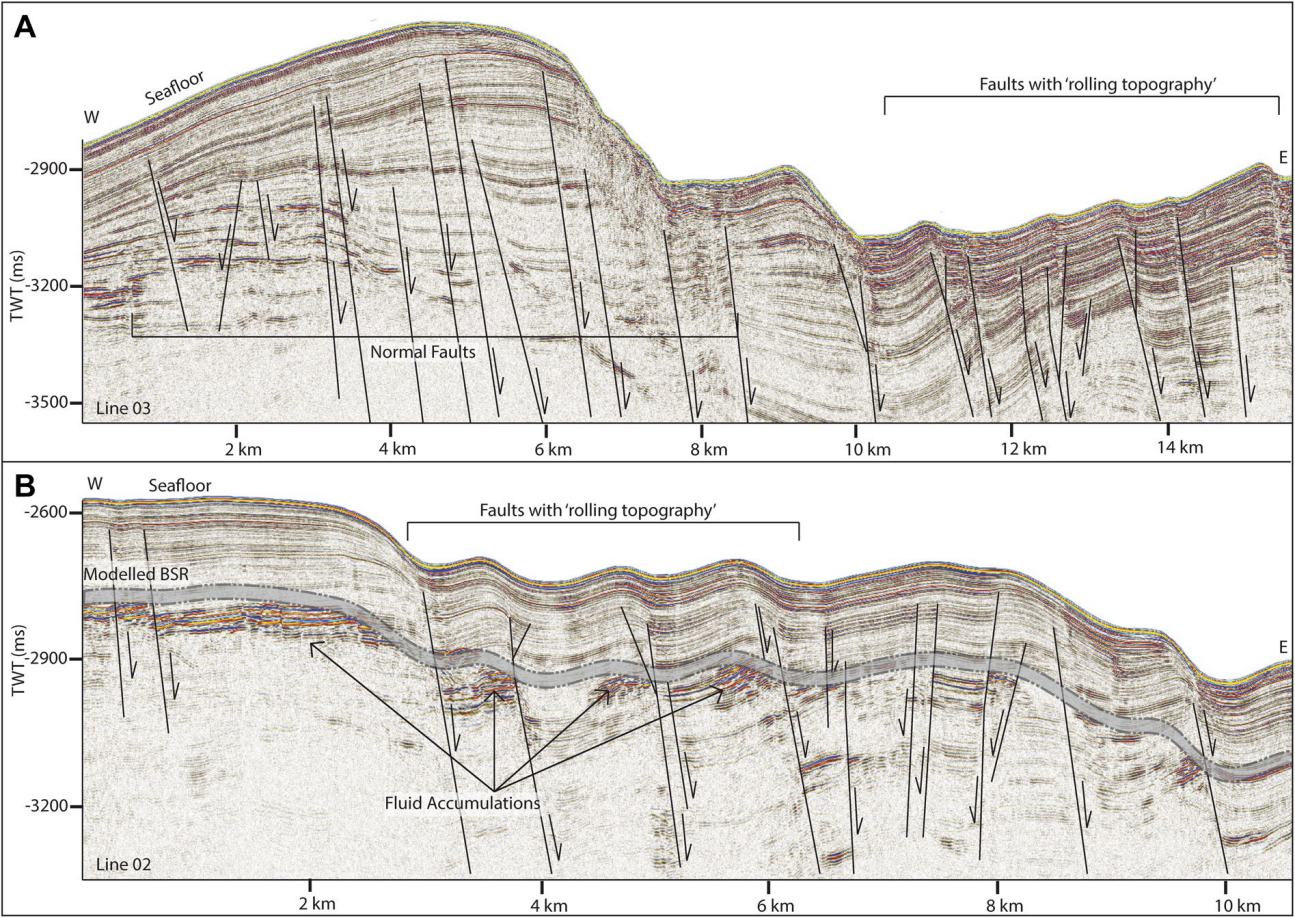
On lines 02 (**B**) and 03 (**A**) we observe a change in sedimentary faulting style. Faults on the main ridge (left, both figures) are normal, while towards the Knipovich ridge, faults begin to (1) interact more with the seafloor, creating a ‘rolling’ topography, (2) have small folds at the upper termination—and on line 01 are associated with fluid accumulations which correspond to the location of the modelled BSR. In addition, faults that are associated with these small folds often appear to have very little reflection offset, a marked steepening towards the seafloor and sometimes a Y shape upper section of the fault.

In addition to major crustal scale tectonic features, we also interpret many sedimentary faults (Figs. 5, 6A, B). These are near vertical, very narrow zones of low amplitude, which offset reflections vertically. In general, almost all the faults imaged are normal faults, offsetting the sedimentary strata by up to 60 ms at depth and decreasing in offset towards the seafloor, interpreted as indicating deformation synchronous with sedimentary deposition^38,39^. The sedimentary faults on lines 02 and 03, however, are associated with a small anticlinal bend at the upper end of the fault plane, and although offset of reflections appears to be very small (~ 20–30 ms offset) corresponding reflections across the fault often have inconsistent amplitude (Fig. 6A, B). These faults often exhibit steepening upwards, or a Y-shaped pattern at the upper section of the fault (Fig. 6A). They are also creating a pronounced rolling topography on the seafloor. These characteristics indicate that these faults are forming under transpression (predominately strike-slip with a compressional component)^40,41^. All sedimentary faults in this data are interpreted as forming in conjunction with deeper tectonic features related to either spreading on the Knipovich Ridge or offset on the MTF.

**Figure 7:**
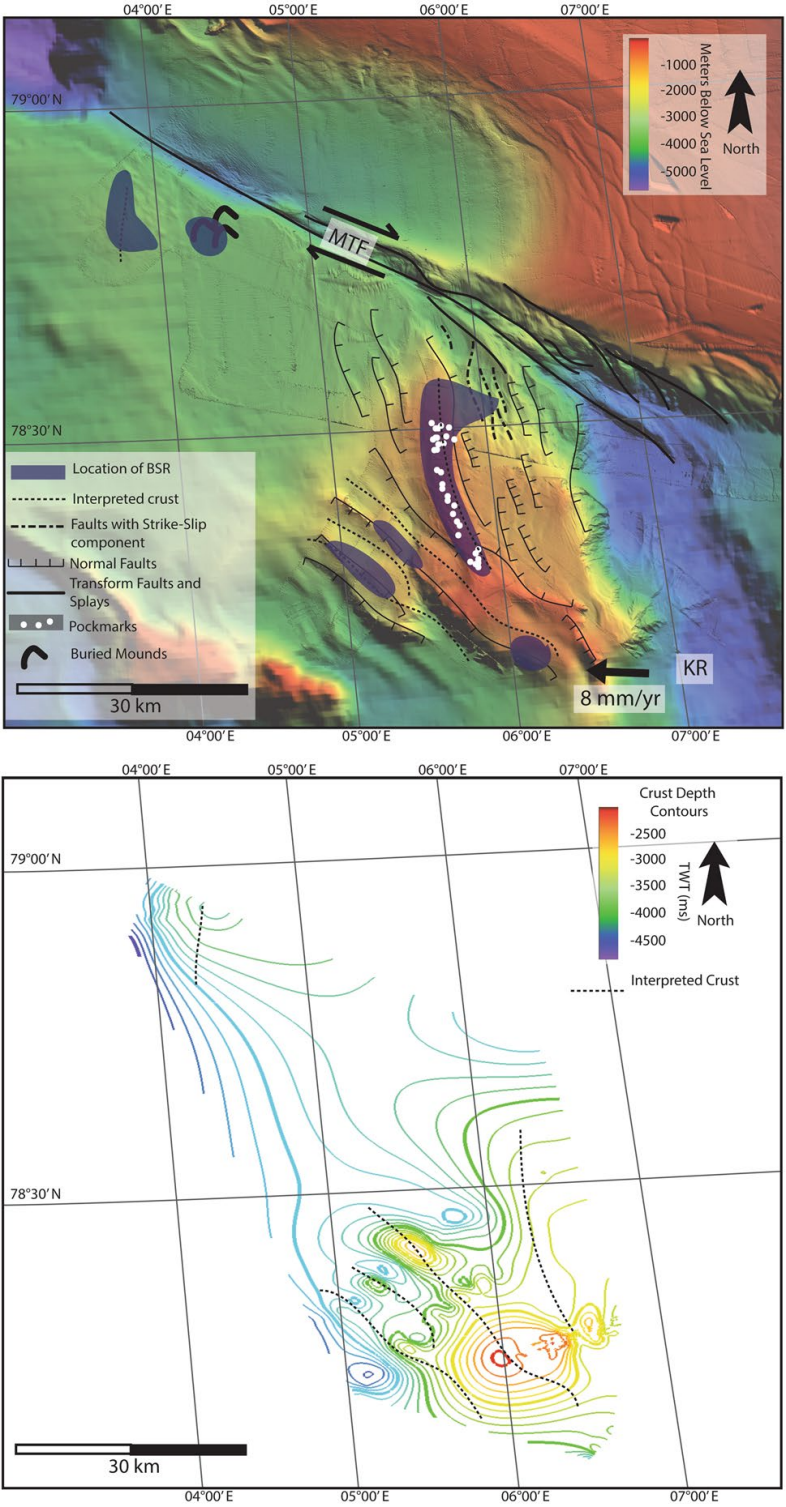
Top: Summary of major fault interpretations (dashed, dotted or solid lines), crustal structure (dashed line), BSR interpretations (blue blobs) and pockmarks (white dots) based on bathymetry and 2D seismic data over the study area. Location of the paleo-transform from Crane et al.^33^. The northern boundary of the study area is the Molloy Transform Fault (MTF), while the eastern boundary is the Knipovich Ridge (KR). Faults are only interpreted on bathymetry where seismic data confirms presence of a fault. Bottom: Crustal depth contours based on 2D seismic lines with crustal structure interpretations. Dashed lines indicate the interpreted crustal structure.

In the 2D seismic data available, we observe significantly more faults when approaching the Knipovich Ridge (Fig. 1). Despite the lack of chronostratigraphic constraints needed to resolve the timing of the fault activity we interpret faults interacting with the seafloor to be indicative of activity as recent as seafloor deposition. It is thus possible to infer that the most recent tectonic activity is focused near the Knipovich Ridge (Fig. 7). This pattern in active faulting focused near the Knipovich Ridge, decreasing with distance away from the ridge is expected in this active MOR tectonic setting. Based on the previous interpretation of faults confined in the sedimentary sequences that are interacting with the seafloor, with a strike-slip component along certain lines, we have also extended these interpretations across the study area using the bathymetry, limiting interpretations to this study area. We observe a small area at the northernmost end of the Knipovich Ridge that has sedimentary faults with a strike-slip component^40^. This area is a small basin-like feature between the Svyatogor Ridge and an interpreted splay fault^25^ related to the MTF. Therefore, we also interpret that the MTF kinematics are influencing at least the northern Svyatogor Ridge (Fig. 7).

### Fluid flow features

**Figure 8:**
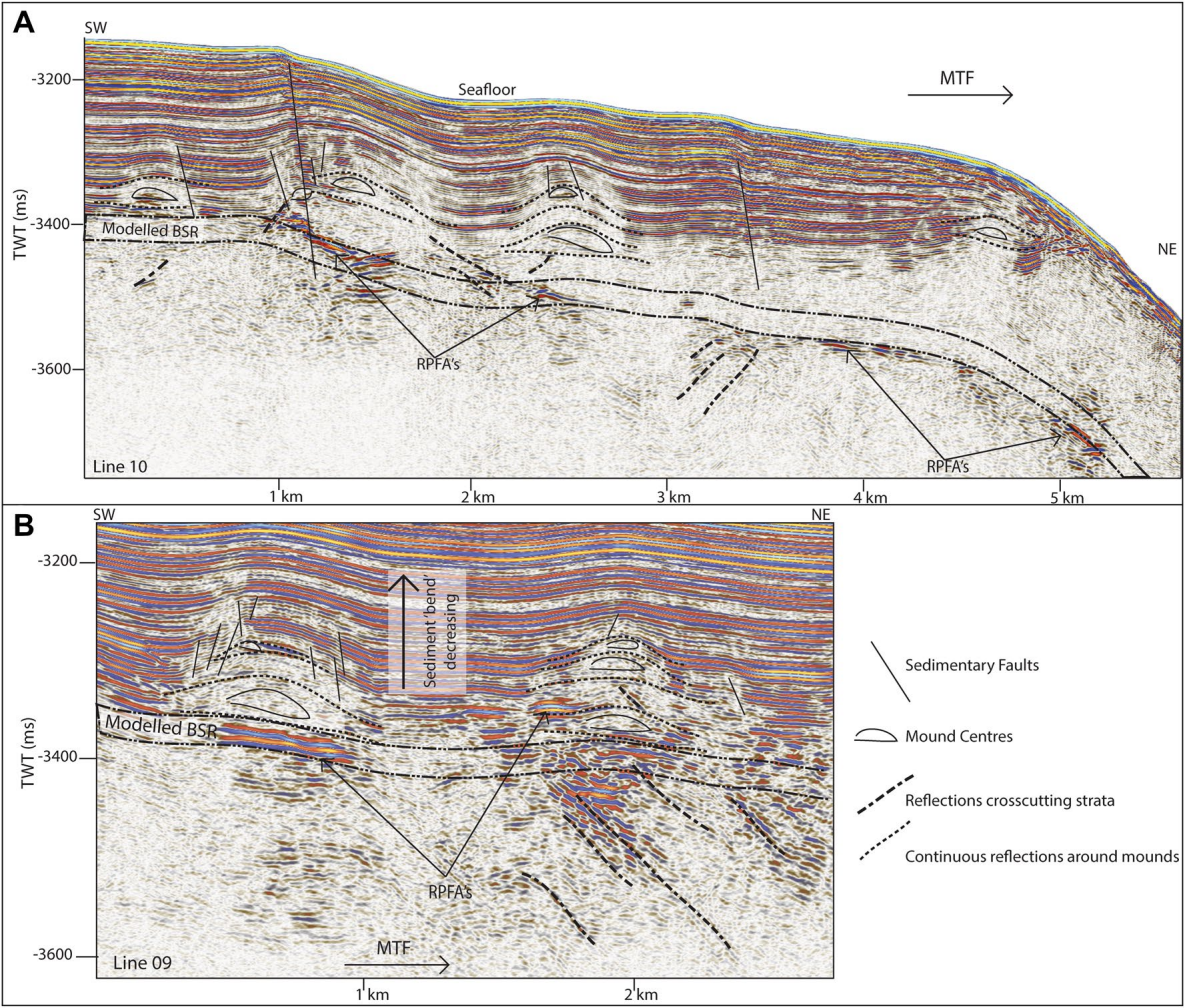
On lines 09 (**B**) and 10 (**A**), mound-like features are interpreted from the western crust high (Fig. 1) to close to the MTF. These mounds have a center, with up domed reflections above. There does not appear to be any onlap or discordant stratigraphy around the mounds, other than the up doming. There are often multiple generations of mounds stacked atop each other, and they are often associated with reverse polarity fluid anomalies (RPFA’s) which sometimes fall into the modelled BGHSZ range. They are also often associated with very small offset sedimentary faults around the top of the mound. Lastly, there are several reflections cross cutting the strata, dipping upwards towards the mounds and RPFA’s.

**Figure 9:**
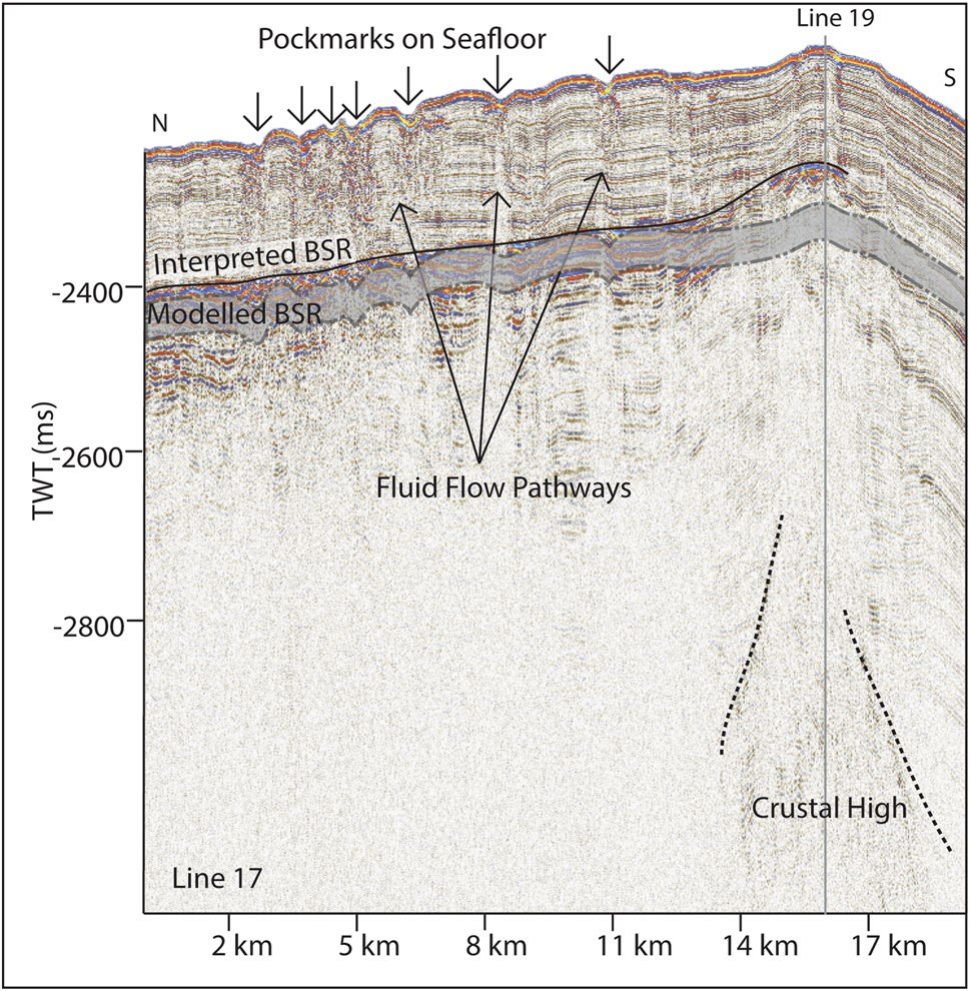
We have interpreted a BSR which is slightly shallower than the modelled BSR in this area. At the southern end of the line, we note crustal structure at depth. At this end of line 17 there are many pockmarks on the seafloor, with vertical, columnar structures of lowered amplitude linking them to the BSR which we interpret as fluid flow pathways.

Reflections characterized by high amplitudes and reversed polarity, appearing to crosscut the stratigraphy while mimicking the seafloor are observed in numerous locations throughout the study area. These reflections correlate on line 19 to the bottom simulating reflection (BSR)—the base of the gas hydrate stability zone—interpreted by Johnson et al.^10^ and Waghorn et al.^11^(Fig. 2). All such reflections in the 2D seismic lines are also interpreted here as BSRs (Figs. 2, 3, 4, 5, 6, 9). The main crest of Svyatogor Ridge contains the thickest zone of high-amplitude anomalies indicating a thick free gas zone, while off the main ridge crest, this zone of high-amplitude anomalies is considerably thinner (Figs. 2, 3). Modeling of the base of gas hydrate stability reveal anomalies in the BSR locations in the region (Figs. 2, 3, 4, 5). One such anomalous BSR is located on the western crustal high, which lies 70 ms above the interpreted crustal structure (Fig. 3). The BSR here is not continuous, however appears to center on the interpreted crustal high, non-uniformly distributed off the crustal high down dip towards the MTF (Fig. 1). Within the sediments between the crustal highs and Knipovich Ridge axis, we also observe sporadic patches of high amplitude reflections denoting fluid anomalies focused on the footwalls of sedimentary faults within small anticlinal structures (Fig. 6B). Towards the MTF in the western section of the study area, there are also sporadic patches of a reflection interpreted to be BSRs (Fig. 8A, B). However, here these reflections are mostly associated with mound structures and lowered amplitudes either above or below the interpreted BSR (Fig. 8A, B). These features are also interpreted as fluid anomaly indicators. In this location we interpret up to three mounds forming consecutively at a single location (Fig. 8A, B), characterized by a seismically transparent ‘core’ and continuous reflections separating the domes. As we only have one transect through these features, we cannot interpret timing nor interpret whether these are distinct domes or a connected system. Domes in this study area are associated with (1) a reverse polarity, high amplitude reflection beneath (Fig. 8A, B) (2) small sedimentary faults with minimal offset around the top of the mounds and (3) crosscutting reflections dipping upwards towards either the high amplitude anomalies or the mound cores (Fig. 8A, B). Here, we interpret these structures as indicators of fluid migration.

The distribution of BSRs across the entire study area is mainly limited to the Svyatogor Ridge crest and areas above crustal highs. Patchy BSRs are also interpreted towards the Knipovich Ridge and towards the MTF (Fig. 7) however there is an apparent strong correlation between the location of crustal highs, related crustal faults (interpreted and assumed) and BSRs in the study area. Modelling the depth of base of GHSZ yields a good fit between the observed BSR and the model output. However, in some instances we observe a systematic mismatch of ~ 20—80 ms between observations and model (Figs. 2, 3, 5). These anomalous BSRs occur over areas where we interpret crustal highs and/or crustal faults. We assert this is an indication that warmer crustal fluids are focused at these locations, because of the crustal structure. Additionally, because the areas of shoaling BSR are located uniquely above crustal faults and highs, and the geothermal gradient data^32,37^ provide a good match at BSR locations without evidence of crustal structure, we assert that the geothermal gradient measurements^32,37^ provide a good regional overview but was collected on a coarse grid so as to not capture localized anomalous heat flux around fault systems.

Figure 9 shows examples of seafloor depression structures above the BSR, which have areas of decreased amplitude and are sometimes associated with faults, interpreted here as pockmarks following Waghorn et al.^15^. U-shaped features with high amplitude bases and low amplitude infill that appear to truncate lower sedimentary horizons on lines 17 and 19 (Figs. 2, 9) are also interpreted as paleo-pockmarks^11^. We observe that all incidences of seepage indicators (paleo pockmarks and pockmarks) are limited to the Svyatogor Ridge and we do not observe such seepage structures anywhere else in the study area (Fig. 7).

## Discussion

### Tectonic regimes

Our interpretation here indicates that the northeastern section of the study area is complex in terms of interactions between the MTF and Knipovich Ridge, where we identify dip-slip faults juxtaposed with oblique slip faulting and faults that appear to have a large strike-slip component (Fig. 6A, B). Splay faulting occurs off the MTF, transitioning into purely dip-slip or possibly oblique-slip faulting with distance southwards^15^. The crustal high interpreted in the west of the study area (Fig. 1), abutting the MTF may indicate the presence of an underlying paleo-detachment fault. It is relatively small compared to other inside corner highs worldwide (i.e. Troodos Ophiolite^42^, the Kane Transform^43^) although this could be an effect of sediment cover smoothing over the crustal topography. The Svyatogor Ridge lies on crust estimated to be between 2.8 and 9.8 Ma^27^, with the potential paleo-detachment in the western section of the study area near to Chron 6, giving an age of < 19.6 Ma^19^ (and 53 km between the two interpreted faults). Although it is not possible to determine the true dip of the feature using 2D seismic data, its apparent dip is approximately 30°, consistent with the dips of other detachment faults in this region.

We also identified anomalous crustal structures southwest of the Svyatogor Ridge (Figs. 4, 5). Peive and Chamov^44^ and Crane et al.^33^ have reported a paleo-transform fault in a similar location as this anomalous crustal structure (Fig. 7). Although the area where we identify shallow crustal structure is not precisely at the previously interpreted paleo-transform, we suggest that the structure here is representative of one block of crust along a strike-slip fault with a slight extensional component. Bathymetry data indicates that if this is indeed a paleo-transform fault, it is asymmetric with the northern flank being considerably elevated compared to the southern flank (Fig. 7). Seismic penetration through the basement structure here does not allow us to discern any faults or features that confirms whether this is representative of a series of fault blocks representing some secondary horizontal stress component along a strike-slip fault; however, the sedimentary sequences appear to have undergone post-depositional deformation above these structures (Figs. 4, 5, 7). Alternatively, these structures, if related to the hypothesized paleo-transform fault, could be representative of splay faults^5^, or duplexes^45,46^ that have formed with continued propagation of the paleo-transform^5^. Splay structures often have large, or entirely, extensional/compressional components compared to the main transform fault segments^5^, which could explain the apparent deformation of sediment along some tectonic lineaments (Fig. 4, 7). Given the uncertainty around the location of the hypothesized paleo-transform, estimating it strikes between 290° and 310° would imply that it is oblique to the spreading axis.

### Heat flow anomalies

Observed BSRs overlying crustal faults in the study area occur shallower than the modelled BSR range at every incidence of interpreted crustal faults (Figs. 2, 3, 5). Even after accounting for uncertainties in the input parameters and errors inherent in the modeling technique (Table 1), the observed BSR is still anomalously shallow at these locations. Similar modelling exercises carried out on the Vestnesa Ridge^38^ find that the largest contributor to error in BGHSZ prediction is the inherent error in pressure estimates^13,36^. Due to the coincidence of these anomalous BSRs with crustal faults, we hypothesize that a likely factor for a slight BSR shoaling here would be localized, fault driven fluid flow that could circulate warmer fluids from depth towards the seafloor. Similar anomalous BSR occurrences driven by advective fluid flow have been reported in the Blake Ridge^47^, Hikurangi Margin^48^, offshore Costa Rica^49^, and Barents Sea^50^. Based on the depths of observed BSRs in the seismic data, we estimate that the geothermal gradient at these locations would need to be ~ 20 °C/km–85 °C/km higher than measured values^32,37^ to account for the mismatch. .

To determine the factors controlling (1) the location of observed BSR and (2) the reason for a discrepancy between observed BSR depth and modelled BGHSZ range, we consider that the geothermal gradient measurements provide a robust regional overview. In most areas of this study, BSR observations fall into the modelled range of BGHSZ, for example, line 03 (Fig. 2B), the mound areas on lines 09 and 10 (Fig. 8A, B) as well as away from the detachment fault on line 17 (Fig. 9). The largest difference between observed and measured geothermal gradients is at the peak of the BSR over the western crustal high (Fig. 3), with a BSR-derived thermal gradient of ~ 204 °C/km versus ~ 120 °C/km interpolated between geothermal measurement stations^32,37^ (Supplementary figure 2). As this area is distal from the spreading axis, we expect that the crust is cooler than proximal to the spreading center. However, as this area is underlain by an interpreted crustal fault it is feasible that this mismatch is caused by the fault and associated warm fluids and advective processes. Even if considered tectonically inactive, an oceanic crustal fault plane which may have undergone partial serpentinization^26^ may still be important for fluid migration as recent work suggests the serpentinization process results in the creation of porosity and thus permeability through fluid-driven mineral fracturing^51^, therefore crustal faults, once formed, may be long-lived pathways for crustal fluid circulation. The geothermal gradient data available at this site was collected on a coarse grid (Supplementary Figure 2) so as to capture the regional trend of the geothermal gradient, whereby this high resolution study provides context to the structure driving heat and fluid flux, and the spatial extent of these processes on an ultra-slow spreading ridge. The results of this geothermal gradient comparison imply that there is active fluid circulation elevating geothermal gradients locally, which may well be linked to ongoing crustal processes such as serpentinization^39^.

### Fluid flow systems

We identify numerous locations with BSRs, or anomalous amplitudes, which we interpret as fluid related anomalies (Figs. 3, 4, 5, 8, 9) that coincide with oceanic crustal high structures (Fig. 2). The crustal high structures correspond with major tectonic features, such as detachment faults, the MTF, the Knipovich Ridge and an interpreted paleo-transform fault^33,44^. Given the available geothermal gradient data^32,37^ and water depth at this study location, as well as bottom water temperature, compared to hotter RTI’s, the Knipovich-MTF RTI is in a regime where abiotic methane linked to serpentinization could well be trapped as gas hydrate in the sedimentary sequences overlying much of the crust.

Ore deposits, such as the massive sulfide deposit at Bent Hill on the Juan de Fuca Ridge^52^, may create mounds on the seafloor associated with hydrothermal fluid circulation and similarly at some cold-seep sites mounds on the seafloor (pingos or pingo-like features) are associated with hydrocarbon gas migration^53^. Pingo-like features can also exhibit similar amplitude effects and often appear as similar domed structures^54^, and so may methane seepage forming carbonate mounds^55^. However ore deposits, pingo-like features in marine settings and carbonate mounds typically form on, or close to the seafloor^54^—here the deformation of sediment above the dome indicates formation in-situ. Underneath some domes, the presence of a high amplitude, reverse polarity reflection (Fig. 3) may indicate their association to the gas hydrate system here. Sediment remobilization^56,57^, or high deposition rates forcing fluid flow^58^ (i.e. overpressure) may also result in mound structures that are not intrinsically linked with formation on the seafloor. With the data available it is difficult to interpret the specific process, however in any of these common mound-forming processes, fluid flow is an important factor in the formation of the features. Therefore, we assert that these dome structures are evidence of sustained paleo-fluid flow.

Waghorn et al.^11^, using 3D seismic data, showed that large-scale crustal faults do indeed underlie the gas hydrate and fluid flow system in the area of the 3D seismic survey, however owing to the lack of additional data, the origin of the methane remains untested. Here, we find that almost every incidence of fluid accumulation, including BSRs, correlates strongly with the presence of crustal basement highs. We have tentatively interpreted that many of these crustal highs are uplifted into their current position due to crustal scale faulting, for example, the detachment faults underlying line 19 or the paleo-transform fault^33^, in the area of lines 15, 16 and 18 (Figs. 1, 7). While abiotic methane is well documented in peridotite-hosted hydrothermal fluid flow systems^24,59^, and on detachment faults^11,14^, Rüpke and Hasenclever^6^ also suggest where ultra-mafic mantle rocks come into close contact with OTF’s, serpentinization is likely. It is striking that in the areas in this study that are above these major tectonic features we do observe fluid and hydrate accumulations (Fig. 7). OBS velocity data^26^ show evidence for partial serpentinization underlying the study area and Klein et al.^60^ and Etiope and Whiticar^61^ indicate that abiotic methane production may occur without macroscale or pervasive serpentinization. Whether serpentinization is occurring at a macroscale or limited to fluid inclusions, crustal faults are still intrinsic in focusing fluid migration into the overlying sediments. Thermogenic methane production in the area would require deep buried sediments containing sufficient organic matter to decompose to hydrocarbon, temperatures above ~ 160 °C and pressures equivalent to burial at 2–4 km. The closest known locations where thermogenic methane production could occur are the Hovgård Ridge and the Vestnesa Ridge^35,62,63^. However, methane migrating from either of these localities would have to traverse significant tectonic lineaments to reach the study area, assuming the presence of the hypothesized paleo-transform^33^ between the Hovgård Ridge and Svyatogor Ridge. Although it is documented that methane accumulations generated in the basin area north of the MTF reach a significant distance towards the transform^64^, there is no evidence that methane is traversing the MTF. A very early gas charge of thermogenic methane might have occurred before the MTF formed, however, the implication of this is that it began ~ 19 Ma, before the western crustal high was offset—approximately when the Fram Strait began opening and sedimentation^27^ initiated, which is unlikely. Lastly, as we have noted the detachment faults imaged on line 19 (Fig. 2) and interpreted crustal structures elsewhere appear to be intrinsic in focusing fluid accumulations. This implies that if methane is not being generated through serpentinization reactions, these structures are in some way connected to a methane source and acting as pathways therefrom. While we would expect microbial methane production to occur widespread over the area containing contouritic deposits, we cannot rule out microbial methane generation in shallow sediments and as noted by Waghorn et al.^11^ shallow penetrating gravity cores did not recover enough gas for isotopic analysis. In the case of microbial methane generation on the Svyatogor Ridge, we still interpret processes pertaining to detachment faulting and proximity to the crust to be intrinsic to the fluid flow system here.

The fluid flow system presented in the study area is unique in that it is not centered on one large drift deposit (i.e. the Vestnesa Ridge^65^, the Blake Ridge^66,67^) nor is it forming on a continental margin with massive sedimentary sequences (i.e. Cascadia margin^68^, Hikurangi margin^69^, Vøring Basin^70^). The system in this study site also appears relatively stable compared to other systems (e.g. Vestnesa Ridge^65^, Blake Ridge^71^)—it is only on the Svyatogor Ridge crest that we find evidence of fluid release—in all other areas of interpreted BSR and free gas accumulations there are no pockmarks or seepage indicators. The dome structures, while indicating the presence of a fluid system do not necessarily imply leakage from a paleo-seafloor; they may have formed in the subsurface, although more data would be required to determine their true nature and origin. Additionally, as the seafloor region cools and subsides with continued seafloor spreading, gas hydrate accumulations charged by abiotic methane will become more stable.

A gas hydrate system occurring on an ultra-slow mid-ocean ridge requires a unique methane source, and, thermal and sedimentary conditions that are generally rare. Elevated geothermal gradients at mid-ocean ridge flanks typically exclude these settings in terms of temperature regime for gas hydrate formation. In addition, thermogenic methane production occurs generally at pressures equivalent to burial to > 2 km depth in the sedimentary column^20^. Sediment columns reaching these depths are highly unlikely at the flank of actively spreading ridges. Therefore, the sedimented ultra-slow spreading ridges of the Arctic are ideal locations to explore and develop a framework for oceanic crust-linked gas hydrate systems. In addition, such Arctic sedimented mid-ocean ridge flanks provide an evolutionary context for prevalent fluid-rock interactions in such settings as fluids produced by deep crustal processes can become trapped in the sedimentary column. Similarly to continental sources of abiotic methane^61^, trapped abiotic methane in the sub-seabed may account for a carbon pool not previously accounted for in global inventories.

## Conclusion

The RTI between the MTF and Knipovich ridge is a unique setting for a gas hydrate system as normally such geologic settings consist of geothermal gradients too warm and minimal to no sedimentary cover for gas hydrate formation and accumulation. The Svyatogor Ridge has a documented fluid flow system which hosts gas hydrate accumulations potentially fueled by abiotic methane. Here, we show that in addition to the Svyatogor Ridge crest, hydrate systems occur across all bathymetric highs in the region underlain by interpreted basement crustal structures. BSRs also overlay large basement crustal structures that may represent a paleo-transform fault immediately south of the Svyatogor Ridge. In addition, some evidence of past fluid flow, in the form of mound structures, are recorded near the western crustal high as well as evidence of sustained fluid flow on the Svyatogor Ridge in the form of paleo and present-day pockmarks. We speculate that the fault plane imaged underneath the western crustal high relates to spreading on the Knipovich Ridge after 19.6 Ma, as the Knipovich Ridge propagated northwards. We therefore propose that these large, crustal-scale, tectonic features are intrinsically involved in supplying methane into the system at this sedimented mid-ocean ridge flank, either through serpentinization of ultra-mafic rock forming methane or as fluid flow pathways dictating the locations of methane and fluid accumulations.

## Supplementary information

Supplementary file1 (DOCX 3 mb)

## Data Availability

The data that support the findings of this study are available from the corresponding author upon reasonable request.
