# Supplementary material for: Crustal processes sustain Arctic abiotic gas hydrate and fluid flow systems
Source: Sci Rep. 2020 Jun 30;10:10679. doi: 10.1038/s41598-020-67426-3 (PMC7326923; doi:10.1038/s41598-020-67426-3)
Supplement: Supplementary file 1 — Supplementary file1 (DOCX 3 mb) [file 41598_2020_67426_MOESM1_ESM.docx]

# Crustal processes sustain Arctic abiotic gas hydrate and fluid flow systems

K. A. Waghorn^1^*, S. Vadakkepuliyambatta^1^, A. Plaza-Faverola^1^, J. E. Johnson^2^, S. Bünz^1^ and M. Waage^1^

^1^CAGE – Centre for Arctic Gas Hydrate, Environment and Climate, Department of Geosciences, UiT – The Arctic University of Norway, Dramsveien 201, 9037 Tromsø, Norway
^2^Department of Earth Sciences, University of New Hampshire, 56 College Road, Durham, New Hampshire 03824, USA

* Corresponding Author: Kate Alyse Waghorn kate.a.waghorn@uit.no

## Supplementary Figures


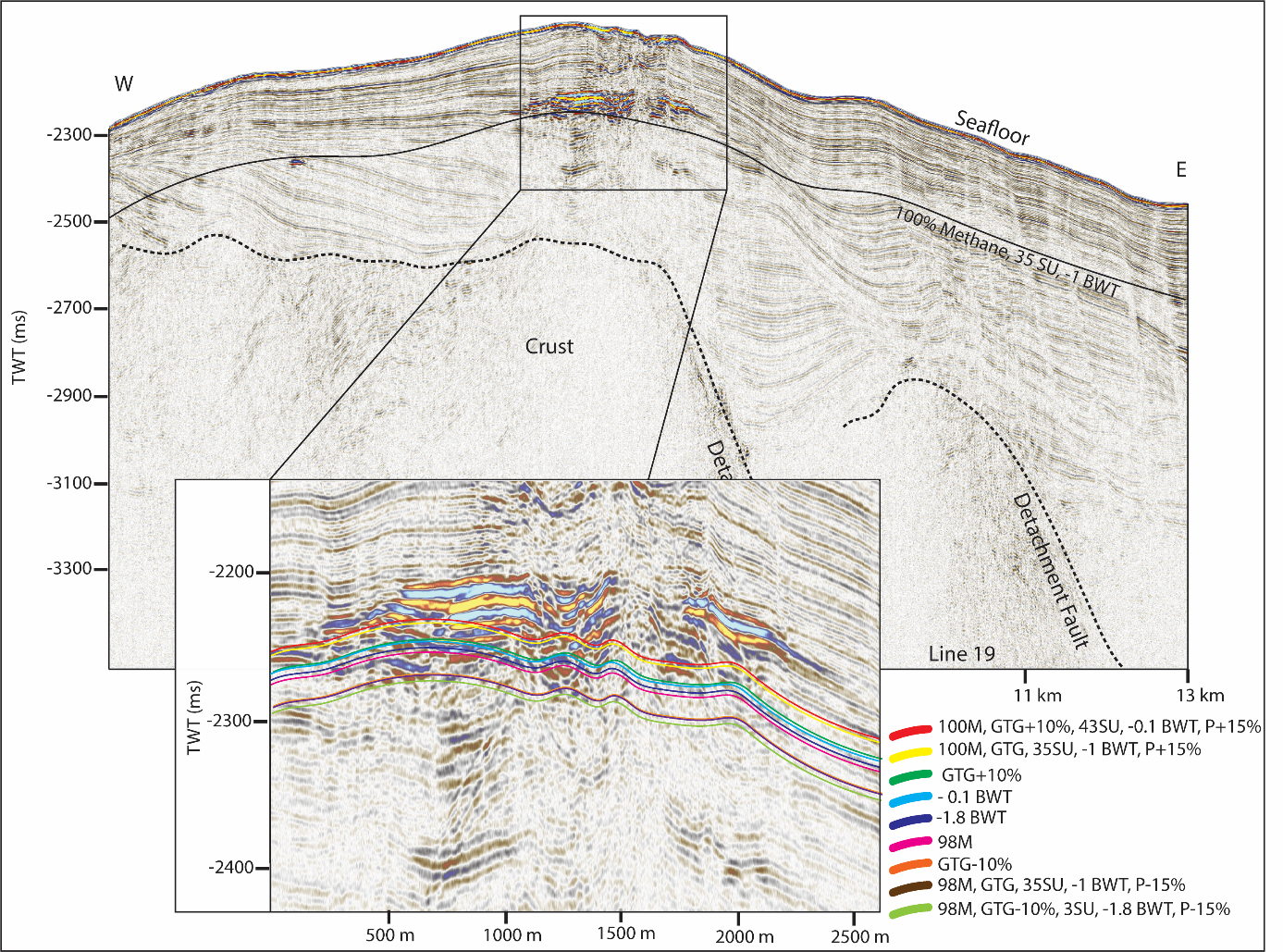


Supplementary Figure 1. Display of some model parameters which developed the range of BSR locations as illustrated on figures in the main article. Baseline values were 100% methane (100M) gas composition, 35 SU salinity, geothermal gradient values ^32,37^ (GTG) and -1°C Bottom water temperature (BWT). Altering values from this baseline shows that 100% Methane, GTG values + 10% error, 43 SU, -0.1°C BWT and +15% error in pressure estimates (red) gives the highest possible location of the BSR, while our baseline variables +15% error in pressure estimates gives a location only slightly lower (yellow). On the other hand, 98% Methane (and heavier hydrocarbons, see methods), GTG values -10% error, 3SU, -1.8 °C BWT and -15% error in pressure gives the lowest possible location (olive green), followed closely by the 98% methane, other baseline variables and -15% error in pressure estimates (brown). Altering other variables keeps the BSR within this range. Therefore, the range between the lowest and highest possible BSR depths after our modelling is the range presented in figures.


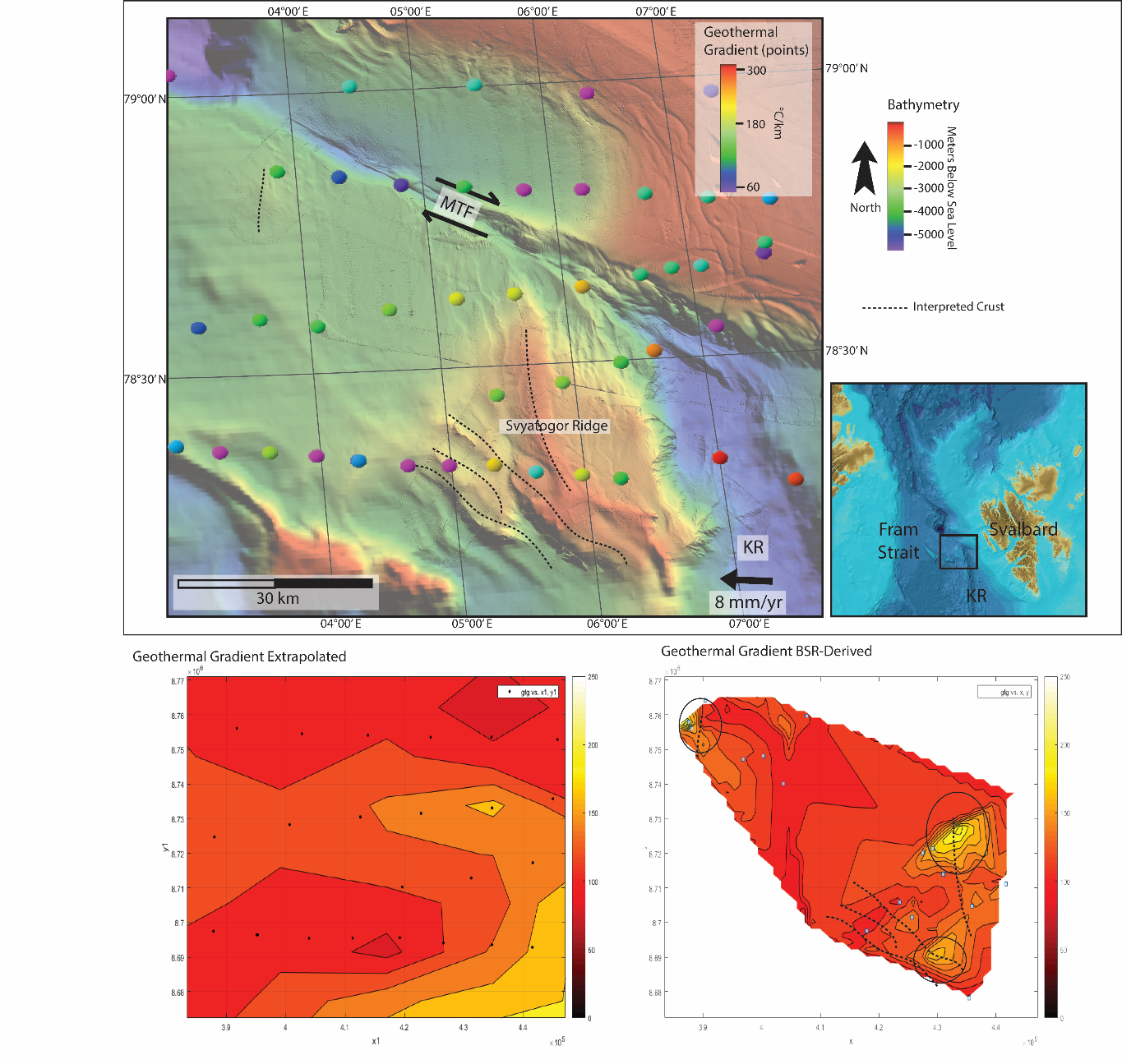


Supplementary Figure 2. Top: Geothermal Gradient measurements from Crane, et al. ^37^ and Crane, et al. ^32^ plotted on bathymetry of this study area. Values in this area range from 63 °C/km to 300 °C/km. From these values, an extrapolated geothermal gradient map (bottom left) covers the study area. Compared to the BSR-Derived geothermal gradient (bottom right), there is overall little difference in the general pattern (higher geothermal gradient towards the Knipovich Ridge, however we note that around areas of crustal faults (black circles, bottom left), there is a higher geothermal gradient than measured values suggest.
